# Generalized Megaesophagus Concurrent With Toxocariasis in a Cat

**DOI:** 10.1155/crve/9504866

**Published:** 2026-10-15

**Authors:** Reza Azargoun, Seyed Mohammad Hashemi-Asl, Rahim Hobbenaghi, Farnaz Malekifard, Borhan Shamsaei, Zahra Nabizadeh

**Affiliations:** ^1^ Department of Internal Medicine and Clinical Pathology Faculty of Veterinary Medicine Urmia University Urmia Iran urmia.ac.ir; ^2^ Department of Surgery and Diagnostic Imaging Faculty of Veterinary Medicine Urmia University Urmia Iran urmia.ac.ir; ^3^ Department of Pathobiology Faculty of Veterinary Medicine Urmia University Urmia Iran urmia.ac.ir

**Keywords:** domestic shorthair cat, esophageal dysfunction, pneumonia, radiology, regurgitation, roundworm

## Abstract

Feline megaesophagus is a rare disorder characterized by esophageal hypomotility and dilation, resulting in regurgitation, weight loss, and occasionally death. This report describes the clinical and paraclinical findings of generalized megaesophagus with a possible contribution of toxocariasis in a kitten. A 2‐month‐old female cat was referred with chief complaints of lethargy, hyporexia, dyspnea, and vomiting/regurgitation. Physical examination of the kitten revealed bradycardia, bradypnea, hypothermia, dehydration, and lung crackles. The most notable laboratory results were leukopenia with severe toxic changes in neutrophils. Radiographic findings were compatible with the diagnosis of generalized megaesophagus. Despite supportive measures, the kitten died the next day. Necropsy revealed diffuse esophageal dilation and *Toxocara* spp. roundworms in the stomach and duodenum. Histopathology showed an ascarid larva in the esophagus, necrosis and metaplasia in the pylorus, and absence of villi and Brunner′s glands in the duodenum. Based on the morphological identification of the adult gastrointestinal nematodes, the final diagnosis was generalized megaesophagus of unspecified classification concurrent with toxocariasis. However, the exact cause of death could not be established. Although respiratory abnormalities raised suspicion of a pulmonary complication, pneumonia was not confirmed. We hypothesized that toxocariasis may have contributed to esophageal dysfunction in this kitten through potential mechanisms such as vomiting, gastrointestinal obstruction, or neurological involvement; however, these mechanisms could not be directly demonstrated. Therefore, parasitic diseases, including toxocariasis, may be considered among potential differential diagnoses or contributing conditions in young cats presenting with megaesophagus, particularly when concurrent gastrointestinal parasitism or related complications are identified. Further studies are required to clarify the possible relationship between toxocariasis and esophageal dysfunction.

## 1. Introduction

Megaesophagus is dilation of the esophagus, characterized by decreased or absent esophageal motility that hinders normal food flow [1]. This condition is classified as congenital or acquired. Congenital megaesophagus is associated with vagal dysfunction, and acquired megaesophagus may be idiopathic or associated with a variety of conditions, including dysautonomia, myasthenia gravis, esophagitis, esophageal obstructions, neoplasia, and diaphragmatic hernias [2]. All types of megaesophagus are rarely diagnosed in cats [3]. Congenital megaesophagus is more common in Siamese and related breeds, whereas acquired megaesophagus can occur in any cat breed [4].

The most common clinical signs include regurgitation, weight loss, poor body condition, altered eating patterns, and difficulty swallowing. Aspiration pneumonia, coughing, respiratory distress, ptyalism, halitosis, nasal discharge, and fever are the most likely complications of megaesophagus. Megaesophagus is typically considered a condition with a poor prognosis, especially when accompanied by aspiration pneumonia [1, 4].

Feline megaesophagus has been understudied, particularly regarding its etiology [5]. In contrast, esophageal dysfunction is rarely described in young kittens and is usually attributed to congenital conditions [6]. To the best of the authors′ knowledge, this case describes a previously unreported concurrent occurrence of generalized megaesophagus and *Toxocara canis* infection in a cat, with an ascarid larva identified within the esophageal wall.

## 2. Case Presentation

A 2‐month‐old female Domestic Shorthair cat weighing 650 g was referred to Urmia University Veterinary Hospital with chief complaints of lethargy, decreased appetite, dyspnea, and persistent vomiting/regurgitation, first observed shortly after weaning. Based on the anxious owner′s account, vomiting could not be differentiated from regurgitation. The cat lived outdoors and had no history of vaccination or deworming.

Physical examination of the kitten revealed bradycardia (heart rate 144 beats per minute), bradypnea (respiratory rate 28 breaths per minute), hypothermia (rectal temperature 37.8°C), and a capillary refill time of 2 s. The animal was underweight, with a body condition score of 2 out of 5. It exhibited pale mucous membranes, moderate dehydration, and mild crackles upon lung auscultation.

The most significant clinical laboratory findings, compared with previously reported hematological values for healthy kittens of the same age [7], were leukopenia (6.6 × 10^3^/ *μ*L vs. 23.68 ± 1.89 × 10^3^/*μ*L), lymphopenia (1.3 × 10^3^/*μ*L vs. 10.17 ± 1.71 × 10^3^/*μ*L), and neutropenia (4.9 × 10^3^/*μ*L vs. 11.0 ± 1.41 × 10^3^/*μ*L), along with an increased hematocrit (38% vs. 33.3*%* ± 0.7*%*). Blood smear examination also revealed severe toxic changes in neutrophils, and structures compatible with hemotropic mycoplasma organisms were observed; however, molecular confirmation was not performed. A plain thoracic radiograph showed soft‐tissue opacity dorsal to the trachea, and the barium esophagogram confirmed generalized megaesophagus (Figure 1). Based on these findings, generalized megaesophagus of unspecified classification was diagnosed. Notably, pupil dilation, dry eyes and nose, third eyelid prolapse, bladder distension, and constipation, which may be suggestive of dysautonomia, were not observed on clinical examination or imaging. The absence of these findings reduced the clinical suspicion of dysautonomia but did not definitively exclude autonomic dysfunction.

**Figure 1:**
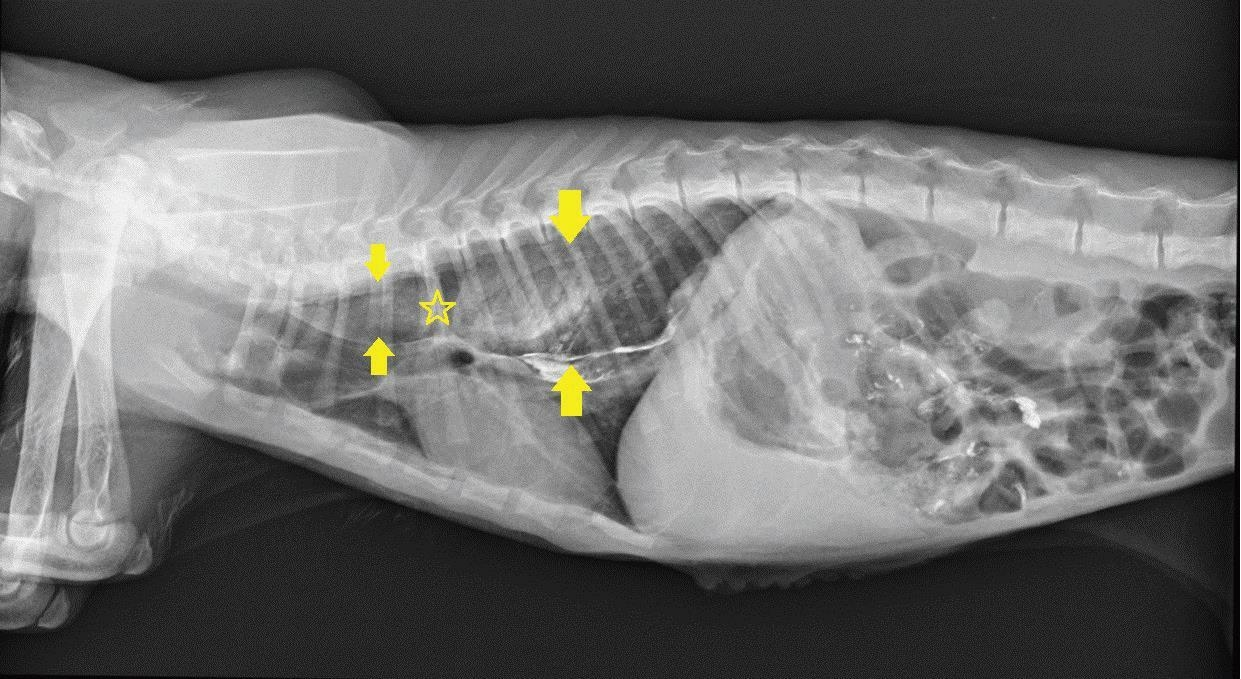
A positive‐contrast esophagram (lateral view) of the cat with generalized megaesophagus and concurrent toxocariasis. The image was obtained immediately after oral administration of barium. The arrows indicate marked dilation of the esophagus along its entire length, with delayed clearance of contrast material from the esophageal lumen. The distended esophagus displaced the trachea and heart ventrally. The asterisk indicates the azygos vein.

Given the patient′s clinical condition, a poor prognosis was anticipated. Supportive measures, including oxygen and fluid therapy, were initiated. Co‐amoxiclav (amoxicillin/clavulanic acid, NORMON, 1000/200 mg vial, 15 mg/kg, intravenously, every 8 h) was selected as empirical antimicrobial therapy because of the risk of aspiration‐associated bacterial complications and concurrent severe gastrointestinal disease. Metoclopramide (OSVE, 10 mg/2 mL ampoule, 0.5 mg/kg, intravenously, every 8 h) was administered as an antiemetic and prokinetic agent rather than as a specific treatment for megaesophagus. Its potential benefit in generalized megaesophagus is uncertain; therefore, the use of metoclopramide in this setting should be interpreted cautiously. In our patient, due to the severity of the clinical condition and the short duration of hospitalization, its clinical effect could not be adequately evaluated. The clinical condition deteriorated rapidly, limiting the opportunity to implement specific nutritional management strategies, such as elevated feeding or assisted nutritional support. The kitten died on the following day.

After obtaining the animal owner′s consent, necropsy was performed immediately, and tissue specimens were collected and placed in 10% buffered formalin. Following standard tissue processing, the paraffin‐embedded blocks were sectioned at five microns and stained with hematoxylin and eosin for histopathological examination under light microscopy.

The main necropsy findings included generalized esophageal dilatation (Figure 2), pulmonary congestion, and white roundworms in the stomach and duodenum. The recovered adult nematodes were morphologically consistent with *Toxocara* spp. and were initially identified as *T. canis* based on their characteristic ascarid morphology (Figure 3), including the presence of three prominent lips, cervical/cephalic alae, and characteristic posterior morphology. Identification was performed by comparison with established morphological descriptions and taxonomic criteria for *Toxocara* spp. [8]. These parasites were associated with partial pyloric obstruction.

**Figure 2:**
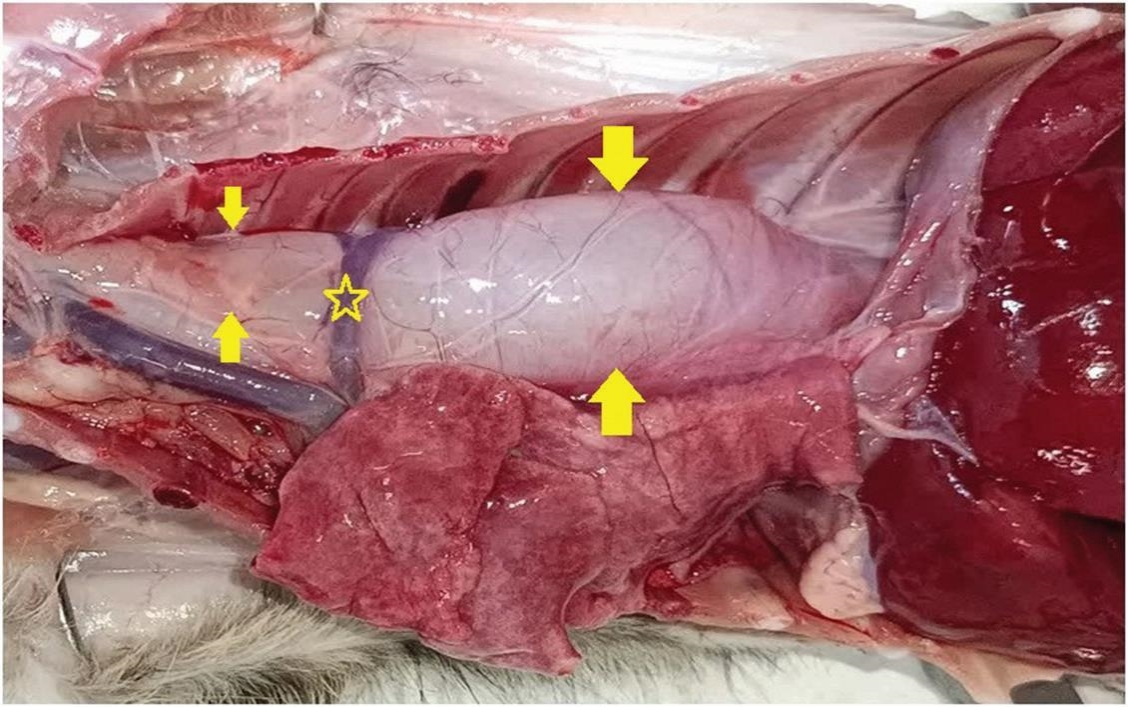
Postmortem examination photograph of the cat with generalized megaesophagus and concurrent toxocariasis. The arrows indicate the esophageal dilation along its entire length. The asterisk indicates the azygos vein.

**Figure 3:**
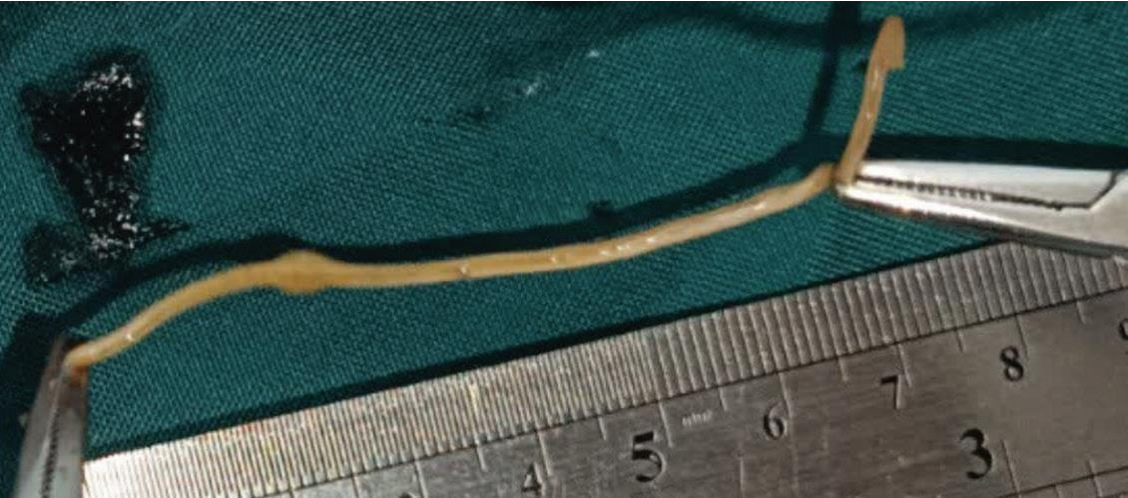
Adult *Toxocara* nematode recovered from the gastrointestinal tract of the cat with generalized megaesophagus and concurrent toxocariasis. The specimen was morphologically identified as *Toxocara canis* based on characteristic adult ascarid features.

Histopathological examination of the esophagus revealed an ascarid larva within the tunica muscularis, with local disruption and degeneration of the surrounding muscle fibers (Figure 4A,B). Based on its morphology and the concurrent recovery of adult *Toxocara* worms from the gastrointestinal tract, the larva was considered compatible with *Toxocara* spp. The larval‐associated lesion was accompanied by extensive submucosal edema and a mononuclear inflammatory cell infiltrate. No fibrosis was observed in the examined section. Only one larva was identified, and no additional larval migration tracks were observed in the examined sections. The neural structures and myenteric plexus could not be adequately evaluated in the available sections. Esophageal epithelial desquamation was also observed, consistent with epithelial injury. Histopathological examination of the pyloric epithelium showed replacement by stratified squamous epithelium, consistent with squamous metaplasia, accompanied by areas of necrosis (Figure 4C). Examination of the proximal duodenum revealed diffuse absence of intestinal villi throughout the examined section, whereas the crypt architecture was preserved, with clearly identifiable crypt lumina and nuclear detail. No significant inflammatory infiltrate was observed. Brunner′s glands were not identified in the examined section. The preservation of cellular and crypt morphology did not suggest autolytic change (Figure 4D).

**Figure 4:**
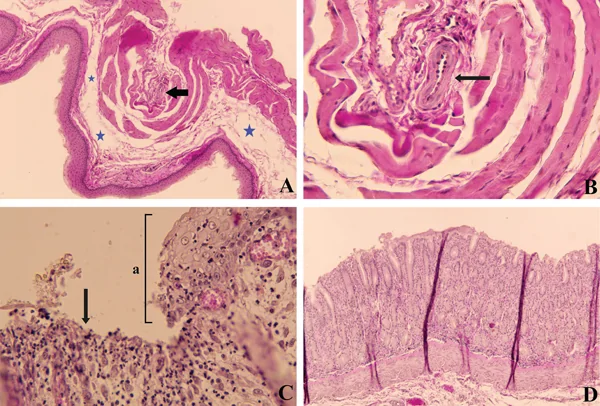
Histopathological findings in the cat with generalized megaesophagus and concurrent toxocariasis. (A) The arrow indicates an ascarid larva in the tunica muscularis of the esophagus, accompanied by extensive submucosal edema (indicated by the asterisks) (×100). (B) The arrow indicates an ascarid larva in the tunica muscularis of the esophagus, with local disruption and degeneration of the surrounding muscle fibers (×400). (C) The pyloric mucosa shows replacement of the normal epithelial lining by stratified squamous epithelium, consistent with squamous metaplasia (indicated by the bracket labeled “a”), with associated areas of necrosis (indicated by the arrow) (×400). (D) The proximal duodenal mucosa shows diffuse absence of intestinal villi throughout the examined section, whereas the crypt architecture is preserved. Brunner′s glands are not identified in the examined section (×100).

Although dyspnea, pulmonary crackles, toxic neutrophil changes, and pulmonary congestion raised suspicion of a pulmonary complication, no radiographic evidence of pneumonia was identified during the clinical evaluation, and pulmonary histopathological examination was not performed. Therefore, pneumonia, including aspiration pneumonia, could not be confirmed, and the cause of death remained undetermined. Based on the clinical, radiographic, and histopathological findings, generalized megaesophagus of unspecified classification concurrent with toxocariasis was considered the final diagnosis.

## 3. Discussion

Cats appear less likely than dogs to develop secondary megaesophagus. This is believed to be due to reduced smooth muscle in the esophagus and to esophageal innervation by autonomic rather than somatic nerves [3]. In the kitten described herein, generalized megaesophagus was diagnosed concurrently with toxocariasis, whereas the adult gastrointestinal nematodes were morphologically identified as *T. canis*. We hypothesized that toxocariasis may have contributed to the development of megaesophagus. Although vertical transmission is well recognized for *T. canis* in dogs, the route of acquisition of *T. canis* infection in this kitten could not be determined. Therefore, maternal transmission cannot be excluded, although evidence supporting this route of infection in the present case is lacking. It is important to note that megaesophagus in kittens can be associated with maternal diseases. For instance, infectious causes such as toxoplasmosis and viral infections may contribute to the development of megaesophagus [9].

Toxocariasis is a zoonotic parasitic disease caused by intestinal roundworms, specifically *T. canis* in dogs and *Toxocara cati* in cats, which serve as definitive hosts [10]. However, toxocariasis in cats is not necessarily due to *T. cati*, as recovery of adult *T. canis* from the intestines of cats following both natural and experimental infections has been documented [11, 12]. Worm burden and prevalence of *Toxocara* spp. infection are highest in kittens. There is a possibility of transplacental and transmammary transmission for *T. canis* [13, 14]. *T. canis* mainly affects animals from birth, presenting clinical signs such as weakness, vomiting, diarrhea, rough coat, growth retardation, cough, abdominal distension, and obstruction [11].

Three potential mechanisms may be considered to explain a possible association between toxocariasis and megaesophagus in this kitten; however, none of these mechanisms were directly demonstrated in the present case.

First, repeated vomiting may theoretically contribute to esophageal dysfunction. In the present case, the owner reported persistent vomiting/regurgitation; however, vomiting could not be reliably distinguished from regurgitation based on the history. Therefore, vomiting itself was neither confirmed nor documented, and its potential contribution to megaesophagus remains hypothetical. Previous observations have suggested that exposure of the esophageal mucosa to gastric contents may affect lower esophageal sphincter function and potentially contribute to esophageal hypomotility [15]. Thus, although repeated exposure to gastric contents may represent a possible mechanism, the available clinical information in this case does not provide sufficient evidence to establish vomiting as a contributing cause of the megaesophagus.

Second, intestinal obstruction and pyloric obstruction may occur in animals harboring roundworms [16]. Gastrointestinal obstruction was documented at necropsy, as the *T. canis* worms were associated with partial pyloric obstruction. However, intragastric pressure was not measured in this patient. Previous experimental evidence indicates that increased intra‐abdominal pressure can adversely affect feline esophageal peristalsis, increase esophageal diameter, and prolong lower esophageal sphincter opening [17]. Therefore, increased abdominal pressure secondary to pyloric obstruction represents a plausible hypothesis in the present case, but this mechanism was not demonstrated.

Third, neurotoxocariasis may theoretically cause neurological dysfunction through larval migration and neuroinflammation [10]. In the present kitten, an ascarid larva was identified within the esophageal tunica muscularis; however, the available histological sections did not permit adequate evaluation of the neural structures or myenteric plexus. Therefore, a neurogenic mechanism could neither be confirmed nor excluded. The previously described association between neuronal injury and megaesophagus in Chagas disease provides a theoretical example of how neuronal dysfunction can result in impaired esophageal motility [18]. However, the Chagas analogy remains hypothetical and cannot be used as evidence for a neurogenic mechanism in this kitten.

The identification of an ascarid larva within the esophageal tunica muscularis was accompanied by local pathological changes, including muscular degeneration, extensive submucosal edema, and mononuclear inflammatory cell infiltration. These findings indicate that the larva was associated with a localized tissue reaction rather than representing an entirely incidental finding. However, only a single larva was identified, and no additional migration tracks were observed in the examined sections. Furthermore, the neural structures and myenteric plexus could not be adequately evaluated histologically; therefore, potential neural injury could not be assessed. Accordingly, although local muscular and inflammatory changes raise the possibility that larval migration contributed to esophageal dysfunction, the available findings do not establish that this focal lesion caused the generalized megaesophagus. Overall, the pathogenesis of megaesophagus in this patient remains uncertain and may have involved multiple factors; however, the present findings do not establish a definitive causal relationship between Toxocara infection and megaesophagus.

The diffuse absence of intestinal villi and the lack of identifiable Brunner′s glands in the examined proximal duodenal section were notable histopathological findings. However, the underlying cause of these abnormalities could not be determined from the available histopathological findings. Although a congenital developmental abnormality could be considered as a possible explanation, there was insufficient evidence to establish a congenital origin. The preserved crypt architecture, clearly identifiable crypt lumina and nuclear detail, and lack of significant inflammatory infiltrates did not support severe generalized mucosal destruction or autolytic change as an explanation for the absence of villi. Similarly, there was insufficient pathological evidence to attribute these findings specifically to malnutrition or parasitic infestation. Therefore, these findings should be interpreted cautiously, and their clinical significance and underlying etiology remain uncertain.

Young kittens are particularly vulnerable to respiratory problems because of their immature lungs [9]. Respiratory abnormalities were also observed in the present kitten, including dyspnea and pulmonary crackles, and pulmonary congestion was identified at necropsy. However, these findings were insufficient to establish pneumonia or aspiration pneumonia, particularly because no radiographic evidence of pneumonia was detected and pulmonary histopathological examination was not performed. Experimental studies have demonstrated pulmonary lesions during Toxocara larval migration; additionally, these larvae may migrate to other tissues of the body [11, 12]. However, pulmonary involvement by Toxocara larvae was not demonstrated in the present case.

Species‐level identification of *Toxocara* larvae in histological sections may be difficult because closely related species such as *T. canis* and *T. cati* may show overlapping morphological characteristics. Molecular methods, including PCR amplification and sequencing of ribosomal and mitochondrial markers, can provide more reliable species‐level discrimination [19]. In the present case, molecular confirmation was not performed. Therefore, although the adult gastrointestinal nematodes were morphologically identified as *T. canis*, the larva identified within the esophageal tissue should be regarded as an ascarid larva compatible with *Toxocara* spp., and its specific identity as *T. canis* cannot be confirmed.

It is worth noting that retrograde migration of roundworms from the small intestine to the stomach can occur, which may irritate the gastric mucosa and result in gastritis. In cats infected with roundworms, gastritis was found in approximately 41% of cases [16]. This event may explain the necropsy and histopathology findings of our study. However, contrary to what would be expected from a parasitic infection, eosinophilia was not observed in the present study. This may indicate the long duration of infection (i.e., from birth). In cats infected with *T. canis* larvae, most animals reach a peak in eosinophilia between Days 14 and 18, followed by a rapid decline toward baseline values [12].

Unfortunately, an anti‐acetylcholine receptor antibody test, which could have provided additional evidence for myasthenia gravis, was not performed in this case. Although the patient did not exhibit persistent generalized weakness (i.e., a floppy cat) or cervical ventroflexion, the absence of these clinical signs does not definitively exclude myasthenia gravis. Similarly, the absence of clinical signs suggestive of dysautonomia reduced, but did not eliminate, the possibility of autonomic dysfunction. In addition, although necropsy and histopathological examination were performed, the myenteric plexuses of the esophagus, vagal or autonomic ganglia, neuromuscular junctions, and other specialized neuromuscular structures were not specifically evaluated. These represent important limitations of this case report. Furthermore, procedures such as bronchoalveolar lavage for cytology and culture could help confirm the diagnosis of suspected pneumonia, but it was not performed due to the patient′s instability.

Another important limitation of this report is the lack of molecular confirmation of the *Toxocara* species. Although the adult gastrointestinal nematodes were morphologically identified as *T. canis*, PCR and sequencing were not performed. Furthermore, the ascarid larva observed within the esophageal wall could not be assigned to *T. canis* at the species level based on histomorphology alone. Molecular analysis of preserved adult and tissue specimens, if available, would have provided stronger evidence for species‐level identification and for determining whether the esophageal larva was genetically related to the adult gastrointestinal parasites.

This case documents an unusual concurrence of generalized megaesophagus, gastrointestinal toxocariasis, and an ascarid larva within the esophageal wall of a young cat. The associated muscular degeneration, submucosal edema, and mononuclear inflammatory infiltration indicate a local tissue reaction; however, the focal nature of the lesion and the inability to adequately evaluate the neural structures preclude establishing a causal relationship between toxocariasis and generalized megaesophagus. Toxocariasis may therefore be considered a possible associated or contributing condition in young cats with megaesophagus, but further investigation is required to determine whether a true pathogenic relationship exists.

## Funding

No funding was received for this manuscript.

## Ethics Statement

Ethical approval was not required for this clinical case report because the report describes a client‐owned animal presented for routine clinical evaluation and diagnostic management and does not involve experimental procedures.

## Consent

Written informed consent was obtained from the owner for the publication of the clinical information, case details, and accompanying diagnostic and pathological images.

## Conflicts of Interest

The authors declare no conflicts of interest.

## Data Availability

The data that support the findings of this study are available from the corresponding author upon reasonable request.
